# Toll-like receptor 7 (TLR7)-mediated antiviral response protects mice from lethal SARS-CoV-2 infection

**DOI:** 10.1128/jvi.01668-24

**Published:** 2025-03-31

**Authors:** Roshan Ghimire, Rakshya Shrestha, Radhika Amaradhi, Lin Liu, Sunil More, Thota Ganesh, Alexandra K. Ford, Rudragouda Channappanavar

**Affiliations:** 1 Department of Veterinary Pathobiology, College of Veterinary Medicine, Oklahoma State University 70729 , Stillwater, Oklahoma, USA; 2 Department of Pharmacology and Chemical Biology, Emory University School of Medicine 12239, Atlanta, Georgia, USA; 3 Department of Physiological Sciences, College of Veterinary Medicine, Oklahoma State University 70729 , Stillwater, Oklahoma, USA; 4 Oklahoma Center for Respiratory and Infectious Diseases, Oklahoma State University 630347, Stillwater, Oklahoma, USA; The Ohio State University, Columbus, Ohio, USA

**Keywords:** SARS-CoV-2, TLR7, interferon, inflammation, myeloid cells

## Abstract

Severe acute respiratory syndrome coronavirus 2 (SARS-CoV-2)-induced impaired antiviral immunity and excessive inflammatory responses cause lethal pneumonia. However, the *in vivo* roles of key pattern recognition receptors that elicit protective antiviral and fatal inflammatory responses, specifically in the lungs, are not well described. Coronaviruses possess single-stranded RNA genome that activates TLR7/8 to induce an antiviral interferon (IFN) and robust inflammatory cytokine response. Here, using wild-type and TLR7-deficient (TLR7^−/−^) mice infected with mouse-adapted SARS-CoV-2 (MA-CoV-2), we examined the role of TLR7 in the lung antiviral and inflammatory response and severe pneumonia. We showed that TLR7 deficiency significantly increased lung virus loads and morbidity/mortality, which correlated with reduced levels of type I IFNs (*Ifna/b*), type III IFNs (*Ifnl*), and IFN-stimulated genes (ISGs) in the lungs. A detailed evaluation of MA-CoV-2-infected lungs revealed increased neutrophil accumulation and lung pathology in TLR7^−/−^ mice. We further showed that blocking type I IFN receptor (IFNAR) signaling enhanced SARS-CoV-2 replication in the lungs and caused severe lung pathology, leading to 100% mortality compared to infected control mice. Moreover, immunohistochemical assessment of the lungs revealed increased numbers of SARS-CoV-2 antigen-positive macrophages, pneumocytes, and bronchial epithelial cells in TLR7^−/−^ and IFNAR-deficient mice compared to control mice. In summary, we conclusively demonstrated that despite TLR7-induced robust lung inflammation, TLR7-induced IFN/ISG responses suppress lung virus replication and pathology and provide protection against SARS-CoV-2-induced fatal pneumonia. Additionally, given the similar disease outcomes in control, TLR7^−/−^, and IFNAR-deficient MA-CoV-2-infected mice and coronavirus disease 2019 (COVID-19) patients, we propose that MA-CoV-2-infected mice constitute an excellent model for studying COVID-19.

**IMPORTANCE:**

Severe coronavirus disease 2019 (COVID-19) is caused by a delicate balance between a strong antiviral and an exuberant inflammatory response. A robust antiviral immunity and regulated inflammation are protective, while a weak antiviral response and excessive inflammation are detrimental. However, the key host immune sensors that elicit protective antiviral and inflammatory responses to severe acute respiratory syndrome coronavirus 2 (SARS-CoV-2) challenge are poorly defined. Here, we examined the role of viral RNA-mediated TLR7 activation in the lung antiviral and inflammatory responses in SARS-CoV-2-infected mice. We demonstrate that TLR7 deficiency led to a high rate of morbidity and mortality, which correlated with an impaired antiviral interferon (IFN)-I/III response, enhanced lung virus replication, and severe lung pathology. Furthermore, we show that blocking IFN-I signaling using anti-IFN receptor antibody promoted SARS-CoV-2 replication in the lungs and caused severe disease. These results provide conclusive evidence that TLR7 and IFN-I receptor deficiencies lead to severe disease in mice, replicating clinical features observed in COVID-19 patients.

## INTRODUCTION

Pathogenic human coronaviruses (hCoVs) cause a variable degree of clinical illness that ranges from mild to moderate to severe respiratory disease in humans. Among the pathogenic hCoVs, severe acute respiratory syndrome coronavirus-2 (SARS-CoV-2), SARS-CoV, and Middle East respiratory syndrome coronavirus (MERS-CoV) have caused case fatality rates of approximately 1, 10, and 35% in humans, respectively (1–8). The SARS and MERS epidemics each caused approximately 800 deaths, whereas the coronavirus disease 2019 (COVID-19) pandemic has resulted in 775 million infections with more than 7 million fatalities worldwide (1, 2, 7, 9). Severe disease caused by SARS-CoV-2 is characterized by high fever, cough, and dyspnea with progression to acute lung injury (ALI), acute respiratory distress syndrome (ARDS), and death in susceptible populations, particularly immunodeficient, elderly, and comorbid individuals (10–18). Patients with fatal pneumonia caused by pathogenic human CoVs exhibit high viral loads compared with those with mild to moderate disease or those infected with low pathogenic coronaviruses (13, 14, 16, 17, 19, 20). Virus-induced direct cytopathic effects and excessive inflammatory cytokine/chemokine responses (also known as ‘cytokine storm’) collectively are believed to cause ALI, ARDS, and fatal pneumonia (10, 21–26). However, the key host factors that facilitate host protection against lung pathology and lethal pneumonia are not completely understood.

Severe COVID-19 is associated with excessive inflammatory cytokine activity and impaired IFN and ISG responses (commonly referred to as ‘dysregulated immunity’) (27–30). SARS-CoV-2 and other hCoVs possess multiple structural and non-structural proteins that antagonize viral RNA-induced IFN/ISG responses (reviewed in [31–34]). While hCoV structural proteins (spike, nucleocapsid, membrane, and envelope proteins) activate TLR2 or TLR4 to primarily induce inflammatory cytokine/chemokine responses (35–43), the CoV single-stranded RNA (ssRNA) genome and its replication intermediate double-stranded RNA (dsRNA) elicit both antiviral and inflammatory responses (38, 44–47). CoV ssRNA primarily stimulates TLR7/8, whereas dsRNA mainly activates MDA5 and/or TLR3 (38, 44–47). Notably, the CoV ssRNA genome is rich in GU/UU nucleotides, which are specifically recognized by TLR7/8 and induce a robust proinflammatory response and a weak type I or type III IFN response (48–50) (51, 52). Conversely, dsRNA activates MDA5/TLR3, resulting in a robust type I or type III IFN response but a relatively weak inflammatory cytokine/chemokine response. Several recent *ex vivo* and *in vitro* studies have shown that SARS-CoV-2-ssRNA-mediated TLR7/8 activation induces IFN/ISG and excessive inflammatory cytokine responses (50, 53). However, the contributions of SARS-CoV-2 ssRNA-induced TLR7 activation to lung antiviral and inflammatory responses, lung pathology, and disease severity are not well understood.

Recent *in vitro* studies using human and murine plasmacytoid dendritic cells (pDCs), myeloid DCs (mDCs), and monocyte-macrophages have shown that TLR7 activation induces robust inflammatory cytokine/chemokine and IFN/ISG responses upon stimulation with SARS-CoV-2 ssRNA or SARS-CoV-2 infection (49, 50, 54). Several elegant human studies have also demonstrated that inborn errors in IFNs, autoantibodies against IFNs, and mutations/deficiencies in TLR3 and TLR7 signaling are associated with severe COVID-19 (53–73). Notably, X-linked recessive TLR7 defects were observed in 1% of COVID-19 patients who presented severe COVID-19. pDCs and other myeloid or myeloid-derived cells isolated from TLR7-deficient COVID-19 patients presented a defective IFN/ISG response (58). These results highlight the critical role of the TLR7-induced IFN/ISG response in host protection. However, given that (i) TLR7 signaling promotes both inflammatory and antiviral responses, (ii) human studies provide correlative evidence for the role of TLR7 activity in severe COVID-19, and (iii) there is an incomplete understanding of the *in vivo* role of TLR7 signaling in lung antiviral and inflammatory responses, the conclusive evidence demonstrating the role of TLR7 signaling in lung viral replication, lung antiviral and inflammatory responses, pulmonary damage, and disease outcomes is lacking. Additionally, given that mice are commonly used animal models for SARS-CoV-2 pathogenesis studies, it is important to define whether TLR7 deficiency in mice replicates the COVID-19 phenotype observed in humans.

Consequently, the primary objective of this study was to elucidate the *in vivo* role of TLR7 signaling in SARS-CoV-2-induced inflammatory/antiviral response and disease outcome. To achieve this goal, we infected wild-type (WT) control and TLR7-deficient mice on a highly susceptible BALB/c background with a mouse-adapted SARS-CoV-2 (MA-CoV-2). WT control and TLR7-deficient mice were evaluated for morbidity and mortality, lung virus titers, antiviral and inflammatory cytokine responses, and pulmonary pathology. We provide conclusive evidence to show that TLR7 signaling is protective during SARS-CoV-2 infection. We further demonstrate that although SARS-CoV-2 ssRNA-induced TLR7 activation elicited a robust inflammatory cytokine/chemokine response, TLR7-mediated antiviral IFN/ISG activity is critical for controlling SARS-CoV-2 replication and host protection.

## MATERIALS AND METHODS

### Sex as a biological variable

We conducted this study using both male and female mice.

### Mice, virus, and infection

The specific pathogen-free male and female control BALB/c mice used in this study were purchased from Charles River Laboratories (strain # 028). TLR7-deficient BALB/c mice re-derived on the Charles River BALB/c background were generated as described previously (44). The mice were maintained in the Animal Resources Animal Care Facility at Oklahoma State University. The mouse-adapted SARS-CoV-2 (N501Y_MA30_, referred to here as MA-CoV-2) strain was a generous gift from Dr. Stanley Perlman (74). Ten- to 16-week-old male and female mice of similar body weights were randomly allocated into two groups (WT control and TLR7^−/^−), with four–five mice per experimental replicate. The mice were lightly anesthetized with isoflurane and challenged with 250 PFU (1 LD50) of MA-CoV-2 via the intranasal route in 50 µL of Dulbecco's modified Eagle medium (DMEM)-only media. For the survival study, the mice were weighed for 10‒14 days and evaluated for clinical signs (respiratory rate, fur condition, posture/movement, and ability to eat and drink). A loss of 25% of the initial body weight or manifestation of severe clinical illness (based on clinical scores) was considered the study endpoint/mortality, and the mice were euthanized by isoflurane overdose, followed by cervical dislocation, as per the AVMA guidelines.

### Virus plaque assay, gene expression, and cytokine quantification

Mice were euthanized at 2 and 5 dpi, and the lungs were perfused transcardially with 10 mL of phosphate-buffered saline (PBS) via the right ventricle. The right lobes of the lungs were homogenized with a tissue bead homogenizer (Fisherbrand Bead Mill 4) in 1 mL of PBS. One part of the lung homogenate was used for SARS-CoV-2 titer determination, and the other part was resuspended in TRIzol for RNA extraction and qPCR.

#### SARS-CoV-2 plaque assay

A 200 µL of 10-fold serially diluted lung homogenate sample was added in duplicate to each well of a 12-well plate seeded with VeroE-hACE2-TMPRSS2 cells and incubated in a 37°C/5% CO_2_ cell culture incubator with gentle rocking every 10 min. After 1 h of incubation, the inoculum was removed and washed with PBS to remove unbound virus particles, and the cell monolayer was overlaid with 1 mL of a 1:1 mixture of 2×DMEM (2× DMEM powder, Corning, Catalog #10-013, 2% FBS, 1% penicillin‒streptomycin, 1% L-glutamine, 1% sodium bicarbonate, 1% sodium pyruvate, 1% nonessential amino acids) and 1.2% agarose. The plates were incubated in a 37°C/5% CO_2_ cell culture incubator for 72 h. After 72 h of incubation, the cells were fixed in 10% paraformaldehyde for 30 min to inactivate the virus. The agarose was removed, and the cells were stained with crystal violet (0.1%). Viral plaques were counted, and the titer was determined.

#### Quantitative PCR (qPCR) for gene expression

Lung homogenate (described above) in TRIzol (TRIZOL, Invitrogen, Catalog #15596026) was used for RNA extraction according to the manufacturer’s instructions. The RNA was treated with RNase-free DNase (Promega, Catalog #M6101) to remove genomic DNA contamination, followed by cDNA synthesis with mMLV reverse transcriptase (Invitrogen, Catalog #28025013). RT-qPCR was performed with PCR Master Mix (Powerup SYBR Green Master Mix, Catalog #A25741) and a real-time PCR system (Applied Biosystems 7500 Fast Real-time PCR System). *Ct* values were determined after normalization of the expression of the gene of interest to that of the housekeeping gene GAPDH, and 2^–∆∆Ct^ values were used to quantify the relative gene expression between control and TLR7-deficient mice.

#### Enzyme-linked immunosorbent assay for inflammatory mediators

Cytokine and chemokine protein level quantification was performed using sandwich enzyme-linked immunosorbent assay (ELISA) from lungs homogenized in PBS supplemented with protease inhibitor cocktail (Millipore Sigma, Catalog #11836170001). The protein levels of IFNβ, IFNλ, TNF, IL1β, and MCP-1 were assayed using commercially available ELISA kits (IFNβ, R&D Systems #DY8234-05; IFNλ, R&D Systems #DY1789B-05; TNF, BD Biosciences #558534; IL1β, R&D Systems #DY401; MCP-1, BD Biosciences #555260) according to the manufacturer’s guidelines. The concentration of proteins was quantified based on a standard curve for each cytokine and expressed as pg/mL.

### Lung cell preparation and flow cytometry studies

To analyze the phenotypes of different immune cell populations in the lungs of control and TLR7-deficient mice, PBS-perfused lung tissue (from the left lobe) was minced into small pieces and treated with tissue digestion buffer containing collagenase-D and DNase-1 as described previously (75). RBC-lysed lung single-cell suspensions were used for immunolabeling. For surface staining, an isolated single-cell suspension prepared from the lung was treated with an Fc block (anti-CD16/32) for 20 min, followed by washing and labeling with fluorochrome-conjugated mouse-specific antibodies: PECy7 α-CD45 (clone: 30-F11, Biolegend, Catalog #103114); FITC α-Ly6G (clone: 1A8, Biolegend, Catalog #127606); PE/PerCp-Cy5.5 α-Ly6C (clone: HK1.4, Biolegend, Catalog #128012); V450 α-CD11b (clone: M1/70, Biolegend: Catalog #101224); PE α-CD11c (clone: N418, Biolegend, Catalog #117308); APC α-Siglec-F (clone: S17007L, Biolegend, Catalog #155507); APC α-Siglec-H (clone: 551, Biolegend, Catalog #129612); and APC α-CD69 (clone: H1.2F3, Biolegend, Catalog #104514). The isolated cells were surface-labeled for markers of neutrophils (CD45^+^ CD11b^+^ Ly6G^hi^), inflammatory monocytes (CD45^+^ CD11b^+^ Ly6c^hi^), plasmacytoid dendritic cells (CD45^+^ CD11b^−^ CD11c^+^ SiglecH^+^), and alveolar macrophages (CD45^+^ CD11b^−^ CD11c^+^ SiglecF^+^). The activation status of inflammatory monocyte macrophages (IMMs) and neutrophils was assessed using the activation marker, CD69. The cells were fixed with Cytofix solution (BD Bioscience, Catalog #554655), and flow samples were acquired on a BD FACS-ARIA-III and analyzed with FlowJo software. Surface staining was carried out by the protocol we previously described (76). All fluorochrome-conjugated antibodies were used at a final concentration of 1:200 in FACS buffer, except the PE/PerCp-Cy5.5 α-Ly6C-labeled antibody, which was used at a concentration of 1:300 in the same buffer used for the study.

### *In vivo* antibody treatment studies

#### Type I interferon receptor (IFNAR) blockade studies

Female BALB/c mice (8–10 weeks old) were infected with a 1 LD50 (250 PFU) of MA-CoV-2 via the intranasal route. The type I IFNAR was blocked with an anti-IFNAR mAb (BioXcell, clone: MAR15A3, Catalog #BE0241, 1 mg/mouse, via the intraperitoneal route), which was administered both before (6–8 h before) and after MA-SARS-CoV-2 infection (3 dpi). A cohort of control and anti-IFNAR mAb-treated mice was used for morbidity and mortality studies. For the virus titer and gene expression studies, the mice were euthanized at 2 and 5 dpi, and the collected lungs were homogenized as described previously for the virus titer assay and gene expression studies.

#### Immune cell depletion studies

Female BALB/c mice (10–12 weeks old) were treated with control, anti-pDC depletion antibody (BioXcell, clone: 927, Catalog #BE0311), or anti-F4/80 depletion antibody (BioXcell, clone: Cl:A3-1, Catalog #BE0206) at a dose of 300 μg/mice via intraperitoneal route 12 hours prior to infection. Following antibody administration, mice were intranasally infected with 1 LD50 (250 PFU) of MA-CoV-2. Mice were euthanized at 2 dpi; lung tissue was harvested as previously described; and cytokine levels were quantified using ELISA. Depletion efficiency was assessed using flow cytometry (Fig. S1).

### Lung histopathology and immunohistochemistry staining

Mice were anesthetized, and the lungs were perfused transcardially with 10 mL of PBS. All four right lung lobes (cranial, middle, caudal, and accessory) were removed and fixed in Zn formalin (Epredia 10% neutral buffer formalin, Thermo Fisher Scientific, Catalog #22-050-104) and embedded in paraffin. The tissue sections were subjected to staining with a modified Harris hematoxylin and eosin (H&E) staining protocol and evaluated in a blinded manner with light microscopy by a board-certified anatomic pathologist. A scoring system was used to assess the extent of edema, fibrin deposition, and lung inflammation in the control and TLR7-deficient mice. The lung pathology scoring criterion was based on the percentage of lung lesions/pathology, with values of 0% (score 0), less than 10% (score 1), 10–39% (score 2), 40–79% (score 3), and more than 80% (score 4) of the examined lung lobe.

For immunohistochemistry staining, paraffin-embedded tissues were heated in a slide warmer (40–50°C) for 30 min and incubated in xylene (Fisher Scientific, Catalog # X3P-1GAL) for 5 min to remove paraffin. The tissue samples were rehydrated in decreasing concentrations of ethanol (100–95−70–50%) for 3 min each. Endogenous peroxidase activity was inhibited by incubating the samples in 3% H_2_O_2_ (Fisher Scientific, Catalog #H324-500) for 10 min at room temperature. The samples were blocked with a CAS block (Thermo Fisher Scientific, Catalog #008120) for 10 min at room temperature. The tissue sections were then incubated overnight at 4°C in a humidity chamber with a primary antibody (rabbit anti-SARS-CoV-2 N protein) (GeneTex, Catalog #GTX635686). The next day, the sections were washed three times with 1× PBS and incubated with a biotinylated goat anti-rabbit secondary antibody (Vector Laboratories, Catalog #BA-1000) for 1 h at room temperature and peroxidase-conjugated streptavidin (Jackson ImmunoResearch, Catalog #016-030-084) for 45 min at room temperature. The samples were developed with ImmPACT DAB EqV (Vector Laboratories, Catalog #SK-4103) and counterstained with hematoxylin (Vector Laboratories, Catalog #H-3404). Immunohistochemical scoring was based on the percentage of SARS-CoV-2 N antigen in the lungs, with values of 0 (absent), 1–25% (score 1), 26–50% (score 2), 51–75% (score 3), and >76% (score 4) of the examined lung lobe.

### Statistical analysis

Statistical analysis was performed with GraphPad Prism Version 9.5.1 (GraphPad Software, Inc.). The results obtained from the study were analyzed with Student’s *t*-test, the Mann‒Whitney test, or one-way analysis of variance (ANOVA), with bar graphs and scatter plot data representing the means +/− SEMs. The statistical significance of weight loss was determined by one-way ANOVA, and survival curves were generated by a log-rank test (Montel–Cox test). A threshold value of *P < 0.05, **P < 0.01, ***P < 0.001, and ****P < 0.0001 was used to assess the statistical significance.

## RESULTS

### TLR7 signaling protects mice from SARS-CoV-2-induced morbidity and mortality

**Fig 1:**
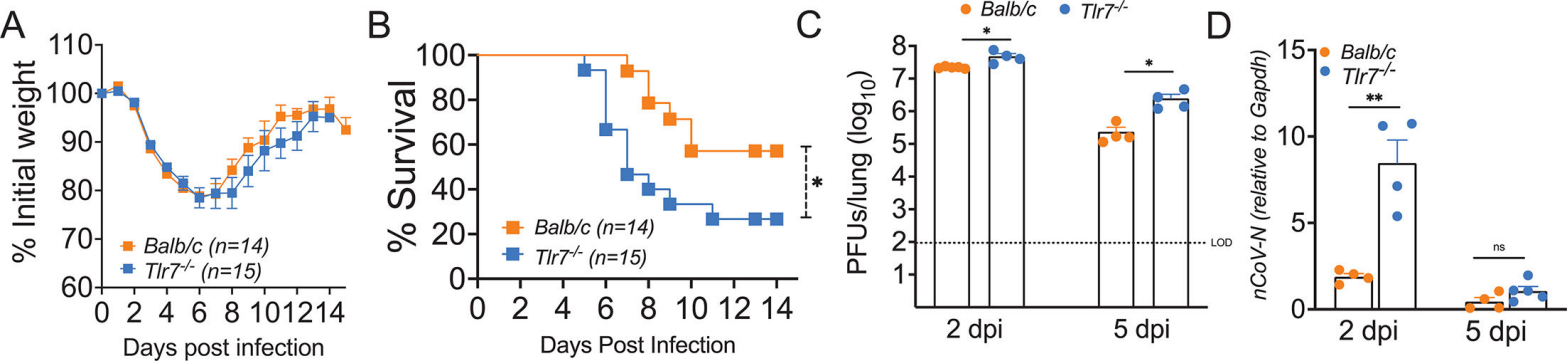
TLR7 signaling protects mice from SARS-CoV-2-induced morbidity and mortality. Ten- to 14-week-old wild-type Balb/c mice and Tlr7^−/−^ mice were infected intranasally with 250 PFU of MA-CoV-2. Percentage initial weight (**A**) and survival (**B**) of mice were monitored for 14 days following infection. The bar graph represents log10 change in SARS-CoV-2 titers (**C**) and subgenomic RNA level (**D**) in control and Tlr7^−/−^ lungs. Data are pooled from three independent experiments with three to five mice/group (A,B) and are representative of three independent experiments with four to five mice/group (C,D). Statistical significance for the survival study was determined using log-rank (Montel–Cox test) (**A, B**) and Student’s *t*-test for (**C, D**) with *P < 0.05, **P < 0.01, and ***P < 0.001.

Loss-of-function TLR7 variants or X-linked TLR7 mutations/deficiencies are associated with severe COVID-19 (53, 77–83). To conclusively establish the role of TLR7 signaling in SARS-CoV-2 pathogenesis and disease outcome, we utilized 10–14-week-old WT control and TLR7-deficient (TLR7^−/−^) mice on a highly susceptible BALB/c background. Male and female control and TLR7^−/−^ mice were infected with 1 LD_50_ dose (250 PFU) of mouse-adapted SARS-CoV-2 (MA-CoV-2) via the intranasal route and monitored for 14 days to assess morbidity (percent initial weight loss) and mortality. Our results revealed that both control and TLR7^−/−^ BALB/c mice lost equivalent weights until day 6 post-infection (Fig. 1A), whereas weight recovery was slow in TLR7^−/−^ mice compared with infected control mice (Fig. 1A), with approximately 75% of TLR7^−/−^ mice ultimately succumbing to death compared with ~40% mortality in control-infected mice (Fig. 1B). Next, we examined the effects of TLR7 deficiency on lung virus titers and viral RNA levels in MA-CoV-2-infected control and TLR7^−/−^ mice at days 2 and 5 post-infection. As shown in Fig. 1C and D, we observed a significant increase in MA-CoV-2 titers on days 2 and 5 post-infection and in subgenomic RNA levels on day 2 post-infection in the lungs of TLR7^−/−^ mice compared with those of infected control mice. These results demonstrated that TLR7 signaling suppresses virus replication and provides host protection during SARS-CoV-2 infection.

### TLR7 promotes antiviral and inflammatory cytokine responses during SARS-CoV-2 infection

**Fig 2:**
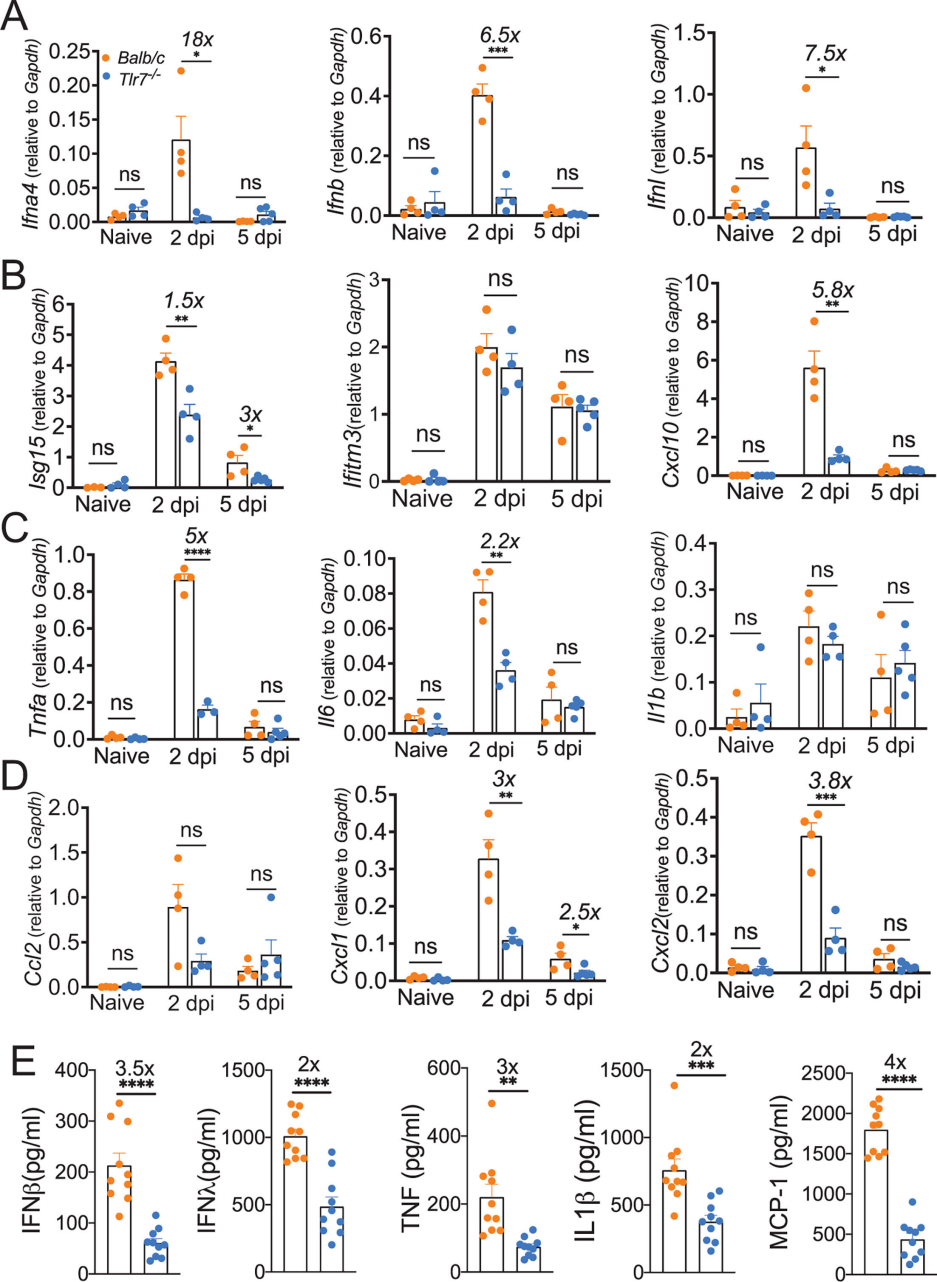
TLR7 signaling is essential for antiviral IFN/ISG response and inflammatory cytokine/chemokine expression in SARS-CoV-2-infected lungs. Ten- to 14-week-old wild-type Balb/c mice and Tlr7^−/−^ mice were intranasally infected with 250 PFU of MA-CoV-2. Lung tissues were harvested on 2 and 5 dpi, and the relative mRNA levels of indicated genes were determined by qPCR. The bar graph shows mRNA levels of IFNs (**A**), ISGs (**B**), inflammatory cytokines (C), and chemokines (**D**) relative to housekeeping gene GAPDH in control and Tlr7^−/−^ mice. The bar graph shows protein levels of indicated cytokines in lungs harvested at 2 dpi in control and Tlr7^−/−^ mice (E). Protein levels are expressed as pg/mL. The results are representative of three independent experiments with *n* = 4–5 mice/group (A–D) or represent data from an experiment with *n* = 10 mice/group (**E**). The symbol “×” above the bar graphs represents ‘fold’ change between groups. Statistical significance was determined using Student’s *t*-test with *P < 0.05, **P < 0.01, ***P < 0.001, and ****P < 0.0001.

*Ex vivo* stimulation of pDCs, DCs, and monocyte–macrophages isolated from the peripheral blood of COVID-19 patients with the TLR7 loss-of-function variant resulted in reduced IFN/ISG levels compared to the cells from control COVID-19 patients (53–55, 80, 81). However, the *in vivo* role of TLR7 signaling in lung IFN/ISG response during SARS-CoV-2 infection is not known. First, we examined the *in vivo* source of IFNs during SARS-CoV-2 infection. Since pDCs and monocyte–macrophages (including alveolar macrophages) are a key source of IFNs during CoV and other RNA virus infections, we depleted pDCs (anti-PDCA1 mAb) and monocyte–macrophages (anti-F4/80 mAb, which targets both AMs and monocyte–macrophages). We found that depletion of pDCs significantly reduced lung IFNβ levels at day 2 post-infection (time of peak IFN response) compared to control-infected mice (Fig. S1A). We also confirmed that both depletion antibodies significantly reduced the percentages of respective target cells in the lungs (Fig. S1B and C). Next, to confirm whether TLR7 deficiency leads to a significantly reduced IFN/ISG response in the lungs and to further define the kinetics of the TLR7-induced IFN/ISG response in SARS-CoV-2-infected lungs, we examined the transcript levels of type I IFNs (*Ifna/b*), type III IFNs (*Ifnl*), and ISGs (*Isg15* and *Cxcl10*) in naïve and MA-CoV-2-infected control and TLR7-deficient lungs. As shown in Fig. 2A and B, we observed a significant increase in IFN and ISG levels in control-infected mice on day 2 post-infection, with a marginal upregulation of some ISGs on day 5 post-infection compared with those in naïve mice. Further assessment of IFNs and ISGs in MA-CoV-2-infected control and TLR7^−/−^ lungs revealed a significant decrease in the mRNA levels of IFNs (*Ifna/b* and *Ifnl*) and ISGs (*Isg15*, *Ifitm3*, and *Cxcl10*) in TLR7^−/−^ lungs compared with those in infected control lungs (Fig. 2A and B). To examine whether IFN transcript expression correlates with their protein levels, we examined IFNβ and IFNλ levels in control and TLR7^−/−^ lungs at 2 dpi. We found that both IFNβ and IFNλ protein levels were significantly reduced in TLR7^−/−^ lungs compared to control-infected lungs (Fig. 2E). These results showed that, similar to *in vitro* PBMC data from TLR7 loss-of-function COVID-19 patients (80), TLR7 signaling is essential for IFN-I/III and ISG induction in SARS-CoV-2-infected lungs.

CoV ssRNA, including the SARS-CoV-2 genome, is abundant in GU/UU-nucleotide sequences (49, 84). This GU/UU nucleotide-rich RNA specifically stimulates TLR7/8 to induce robust proinflammatory cytokine and chemokine production (49, 50, 84). We recently showed that a ssRNA mimic (a TLR7 agonist) induces robust proinflammatory cytokine production compared with dsRNA (a TLR3/MDA-5 agonist) and DNA mimics (a TLR9 agonist) (76). Therefore, to assess whether TLR7 activation by viral RNA facilitates robust inflammatory cytokine and chemokine production in MA-CoV-2-infected lungs, we examined the mRNA levels of key inflammatory cytokines and chemokines in the lungs. Quantitative PCR analysis revealed increased mRNA levels of proinflammatory cytokines and chemokines (*Tnf*, *Il6*, *Il1b*, *Ccl2*, *Cxcl1*, and *Cccl2*) in SARS-CoV-2-infected lungs compared with those in naïve lungs on days 2 and 5 post-infection (Fig. 2C and D). Additionally, similar to the IFN/ISG expression levels, the mRNA levels of several proinflammatory cytokines and chemokines in the MA-CoV-2-infected TLR7-deficient lungs were significantly lower than those in the infected control lungs (Fig. 2C and D). Further examination of protein levels of inflammatory cytokines (TNF and IL-1β) and chemokines (MCP-1) at day 2 post-infection showed significantly reduced levels of these mediators in TLR7^−/−^ lungs compared to control lungs (Fig. 2E). These results demonstrated that TLR7 signaling not only promotes IFN/ISG expression but also facilitates SARS-CoV-2-induced robust inflammatory cytokine and chemokine responses in the lungs.

### TLR7 deficiency leads to differential myeloid cell accumulation in SARS-CoV-2-infected lungs

**Fig 3:**
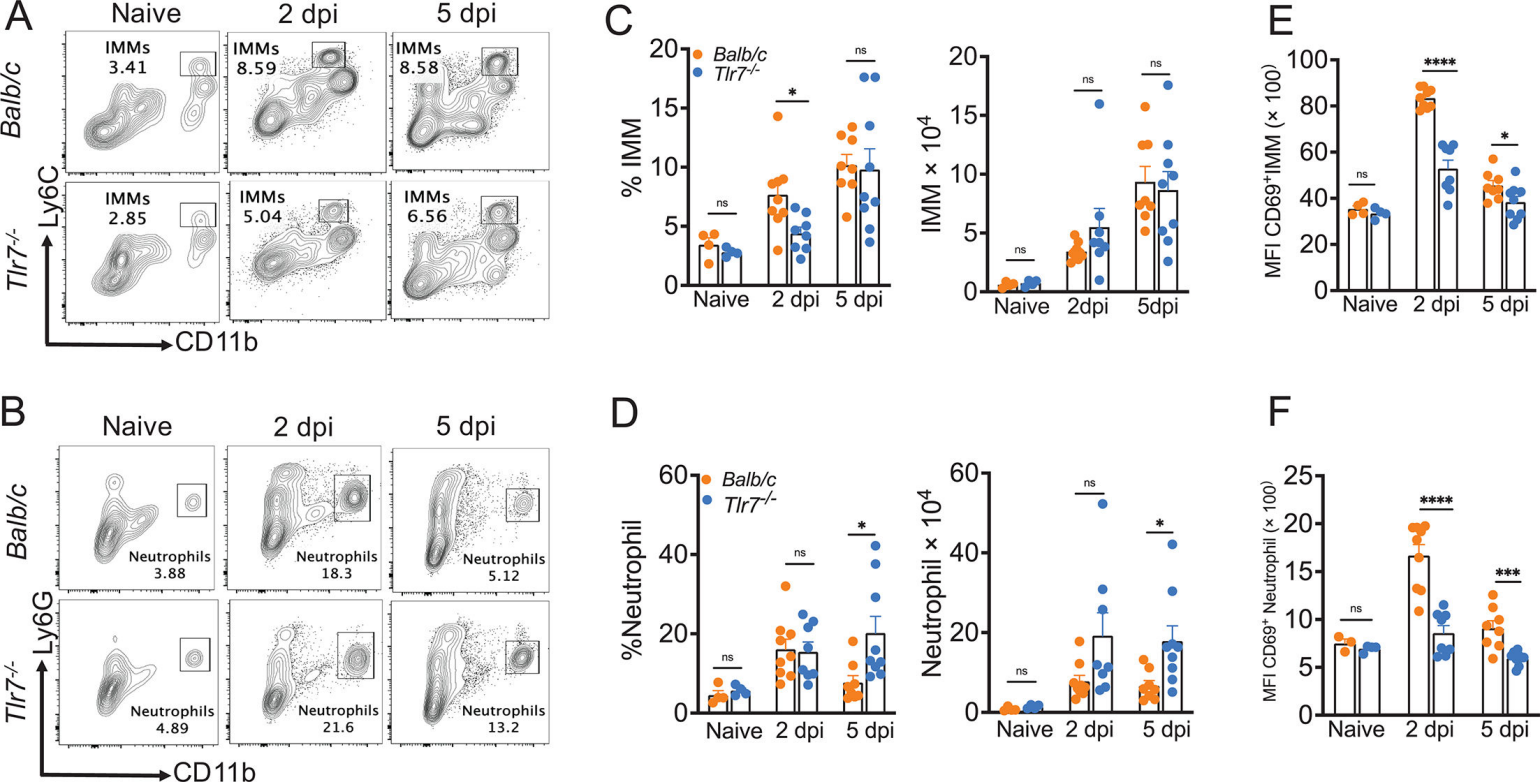
TLR7 deficiency causes differential myeloid cell accumulation in SARS-CoV-2-infected lungs. Lung tissues harvested from Balb/c and Tlr7^−/−^ mice challenged with 250 PFU of MA-CoV-2 were analyzed for infiltrating immune cells at days 2 and 5 post-infection. (**A**) Representative FACS plot (upper left) shows the percentage of IMM in lungs at 2 and 5 dpi. (**B**) Representative FACS plot (lower left) shows the percentage of neutrophils in lungs at 2 and 5 dpi. (**C**) The bar graph shows the percentage and total IMM in the lungs at 2 and 5 dpi. (**D**) The bar graph shows the percentage and total neutrophils in the lungs at 2 and 5 dpi. (**E**) Bar graph showing MFI of CD69 expression on IMM cells. (**F**) Bar graph showing MFI of CD69 expression on neutrophils. Data are pooled from two independent experiments with *n* = 4–5 mice per group. Statistical significance was determined using Student’s *t*-test with *P < 0.05, **P < 0.01, ***P < 0.001, and ****P < 0.0001.

Excessive myeloid cell activity, specifically monocyte‒macrophage and neutrophil responses, cause severe hCoV disease (26, 85–90). Robust IFN-I and inflammatory cytokine/chemokine responses drive myeloid cell influx and inflammatory activity (28, 44, 91). To define the effect of TLR7 deficiency on immune cell accumulation and examine whether a reduced antiviral and inflammatory cytokine/chemokine response in TLR7-deficient mice leads to altered myeloid cell responses in the lungs, we euthanized MA-CoV-2-infected control and TLR7-deficient mice at 2 and 5 dpi. PBS-perfused collagenase/DNAse-digested lungs were analyzed for myeloid cell infiltration by flow cytometry. As shown in Fig. 3A through D, we observed a significant increase in inflammatory monocyte‒macrophage and neutrophil accumulation in SARS-CoV-2-infected lungs compared with that in naïve mouse lungs. Furthermore, TLR7-deficient lungs presented a reduced percentage of inflammatory monocyte‒macrophages at 2 dpi, but the total number of these cells did not differ between infected control and TLR7-deficient lungs on either day post-infection (Fig. 3A and C). Conversely, we observed a significant increase in the percentage and total number of neutrophils in TLR7-deficient lungs at 5 dpi (Fig. 3B and D). Next, to define the functional correlates of myeloid cells, we examined the activation status of these immune cells in control and TLR7-deficient lungs. Our results showed significantly reduced CD69 levels in monocyte–macrophages and neutrophils of TLR7^−/−^ lungs compared to control mice at day 2 post-infection (Fig. 3E and F). These results show that the loss of TLR7 signaling results in differential accumulation of myeloid cells with a specific increase in neutrophils in TLR7^−/−^ lungs, although both monocyte–macrophages and neutrophils are less activated in TLR7^−/−^ compared to control lungs.

### TLR7 signaling protects mice from SARS-CoV-2-induced lung pathology

**Fig 4:**
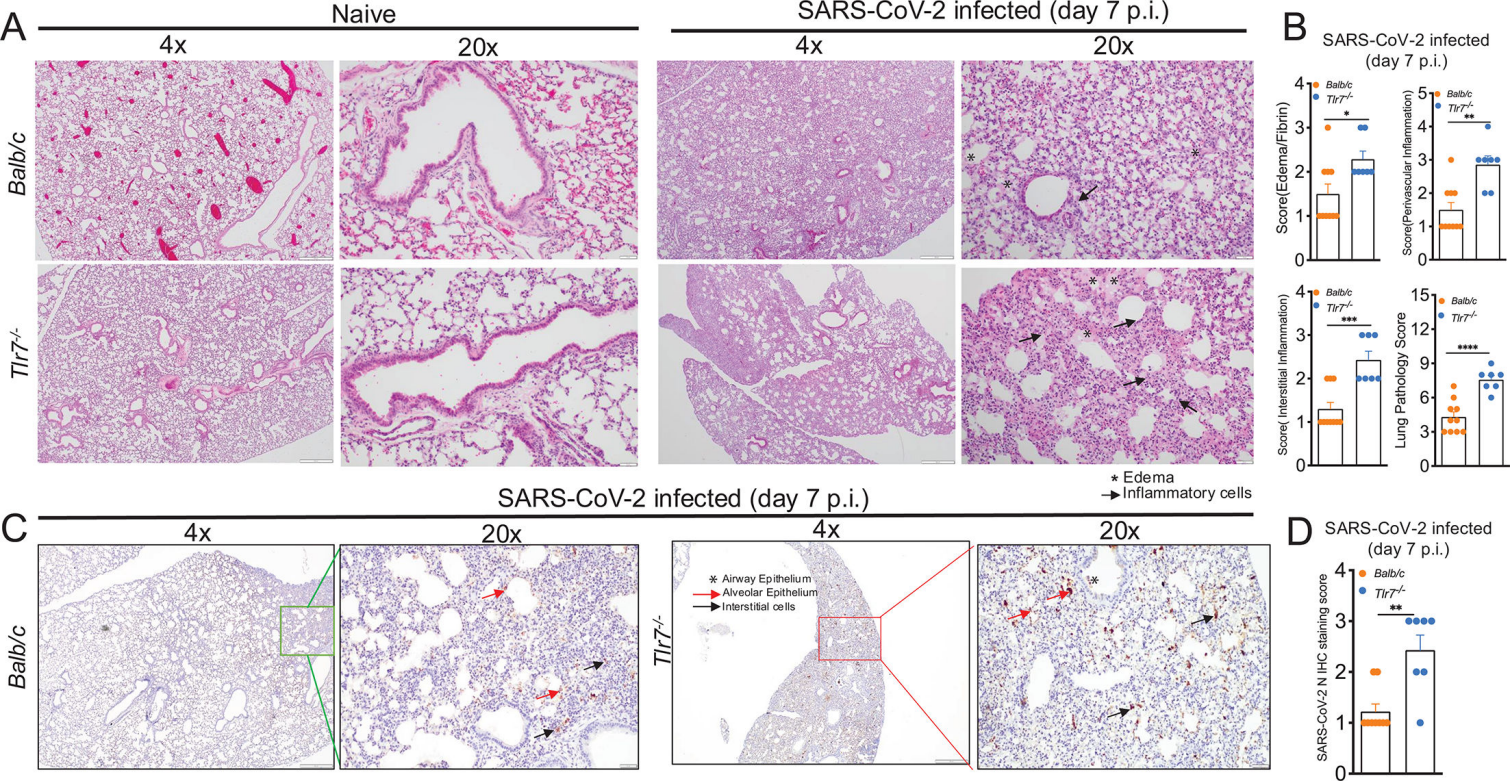
TLR7 deficiency promotes lung pathology during SARS-CoV-2 infection. MA-CoV-2-infected Balb/c and Tlr7^−/−^ mice were euthanized at 7 dpi. PBS-perfused lungs collected in Zn formalin were stained with hematoxylin and eosin to assess lung lesions. (**A**) Representative photomicrographs showing edema and inflammatory cell infiltration in the lungs. (**B**) Summary histology scores for edema/fibrin, interstitial inflammation, perivascular inflammation, and cumulative lung pathology score. (**C**) Immunohistochemical analysis for SARS-CoV-2 N antigen in lungs at 7 dpi. (**D**) Summary immunohistochemical scores for SARS-CoV-2 N antigen in lungs. Each point in the scatter plot represents individual mice (*n* = 10 for Balb/c and *n* = 7 for Tlr7^−/−^ mice). Statistical significance was determined using Student’s *t*-test with *P < 0.05, **P < 0.01, ***P < 0.001, and ****P < 0.0001.

TLR7 deficiency is associated with severe COVID-19 (53). However, owing to the lack of lung autopsy or biopsy samples from TLR7-deficient COVID-19 patients, the effect of TLR7 signaling or a lack thereof on lung inflammation and pathology is not known. Therefore, to gain insight into the lung pathology of SARS-CoV-2-infected control and TLR7^−/−^ hosts, we examined the lung histopathology of control and TLR7-deficient mice infected with 1 LD50 of MA-CoV-2. Tissue sections from PBS-perfused lungs collected at 7 dpi were stained with hematoxylin and eosin to assess lung inflammation and pathology. The lung lesions were graded on a scale of 0 to 4 (0 being normal and 4 being severe) on the basis of edema, fibrin deposition, and interstitial inflammation. Our results revealed a marked increase in lung pathology in infected control and TLR7^−/−^ mice compared with that in naïve mice (Fig. 4) and significantly increased alveolar/bronchial edema and fibrin deposition in TLR7^−/−^ lungs compared with control-infected lungs (Fig. 4A and B). Additionally, there was a significant increase in inflammatory cells, as characterized by the accumulation of mono- and polymorphonuclear cells in the perivascular and interstitial spaces, in TLR7^−/−^ lungs compared with those in control lungs (Fig. 4A and B). Furthermore, interstitial septal thickening was more pronounced in TLR7^−/−^ lungs than in WT lungs, suggesting structural changes associated with TLR7 deficiency (Fig. 4A). Overall, lung pathology scores were significantly greater in infected lungs than in naïve lungs, with infected TLR7-deficient lungs showing more pronounced lung lesions than infected control lungs (Fig. 4B).

Next, to elucidate whether TLR7 deficiency results in differential infection and tissue distribution of the virus, we performed an immunohistochemical examination of the SARS-CoV-2-N antigen in infected control and TLR7-deficient lungs. While we did not observe any difference in viral antigen distribution in control or TLR7^−/−^ lungs at 2 dpi (data not shown), we detected significantly increased viral antigen abundance at 7 dpi, primarily in macrophages (alveolar and interstitial) and epithelial cells (type II pneumocytes and bronchial) in TLR7^−/−^ lungs compared with control lungs (Fig. 4C and D). These results showed that TLR7 deficiency delays SARS-CoV-2 clearance and/or promotes persistent infection of epithelial and myeloid cells in infected lungs.

### Loss of IFNAR signaling leads to lethal SARS-CoV-2 infection and severe lung pathology

**Fig 5:**
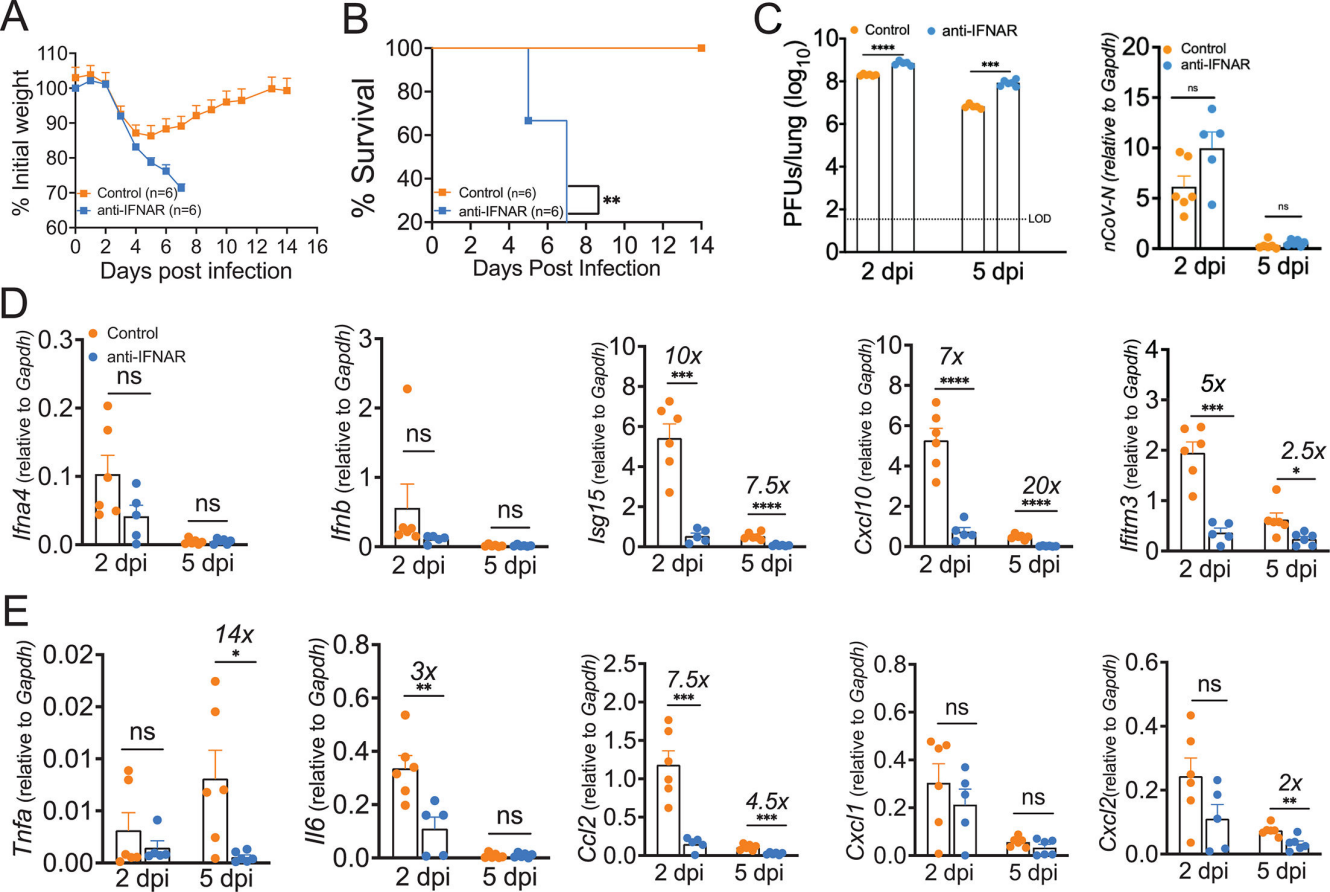
Interferon-I signaling protects mice from SARS-CoV-2-induced morbidity and mortality. Twelve- to 14-week-old wild-type Balb/c mice were treated with control or anti-IFNAR antibody and infected intranasally with 250 PFU of MA-CoV-2. Percentage initial weight (**A**) and survival (**B**) of mice were monitored for 14 days following infection. The bar graph (**C**) represents log10 change in virus titers and subgenomic RNA level in lungs of control and anti-IFNAR-treated mice. The bar graph shows mRNA levels of indicated IFNs and ISGs (**D**) and inflammatory cytokines/chemokines (E) relative to housekeeping gene GAPDH in lungs of control and anti-IFNAR-treated mice. Data are derived from six mice/group. The symbol “×” above the bar graphs represents ‘fold’ change between groups. Statistical significance was determined using log-rank (Montel–Cox test) (**A, B**) and Student’s *t*-test (**C, D, E**) with *P < 0.05, **P < 0.01, ***P < 0.001, and ****P < 0.0001.

Our results showed TLR7 deficiency leads to significantly reduced levels of IFNs, IFN-stimulated genes, and inflammatory mediators (Fig. 2), all of which correlated with increased disease severity (Fig. 1). These data are correlative and do not conclusively prove that reduced IFN/ISG responses cause severe disease in TLR7-deficient mice. Therefore, to establish that reduced IFN/ISG responses observed in TLR7^−/−^ mice contribute to severe disease, we blocked type-I IFN signaling (IFNAR blocking antibody) in SARS-CoV-2-infected BALB/c mice. For these studies, 8–10-week-old wild-type BALB/c mice were either control-treated or treated with anti-IFNAR mAb before (−8 h) and after (3 dpi) infection with 1 LD50 of MA-CoV-2. Control and anti-IFNAR mAb-treated mice were monitored for weight loss and survival for 14 days. Our results illustrate that control BALB/c mice initially lost approximately 15% of their initial body weight until day 5 post-infection and then recovered, whereas the anti-IFNAR mAb-treated mice lost more than 25% of their initial body weight, with 100% of the mice succumbing to death by day 7 post-infection (Fig. 5A and B). We also examined virus titers and subgenomic RNA levels in the lungs of control and anti-IFNAR mAb-treated mice. As shown in Fig. 5C, we detected a significant increase in the viral titer at 2 and 5 dpi in the anti-IFNAR mAb-treated lungs compared with the control lungs. The subgenomic RNA level was also greater at 2 dpi in anti-IFNAR mAb-treated lungs than in control lungs, but the difference was not statistically significant (Fig. 5C). Additionally, we examined the mRNA levels of IFNs/ISGs and inflammatory cytokines/chemokines in control and anti-IFNAR mAb-treated lungs. We detected a significant decrease in the mRNA levels of ISGs (*Isg15*, *Cxcl10,* and *Ifitm3*) at both 2 and 5 dpi in the anti-IFNAR mAb-treated mice compared with the control mice (Fig. 5D), whereas the *Ifnα*/*β* transcript levels were comparable between the two groups (Fig. 5D). We also found a significant decrease in the mRNA levels of inflammatory cytokines and chemokines (*Tnf*, *Il6*, and *Ccl2*) at one or both time points, although the mRNA level of *Cxcl1* did not differ on either day post-infection in either group (Fig. 5E).

**Fig 6:**
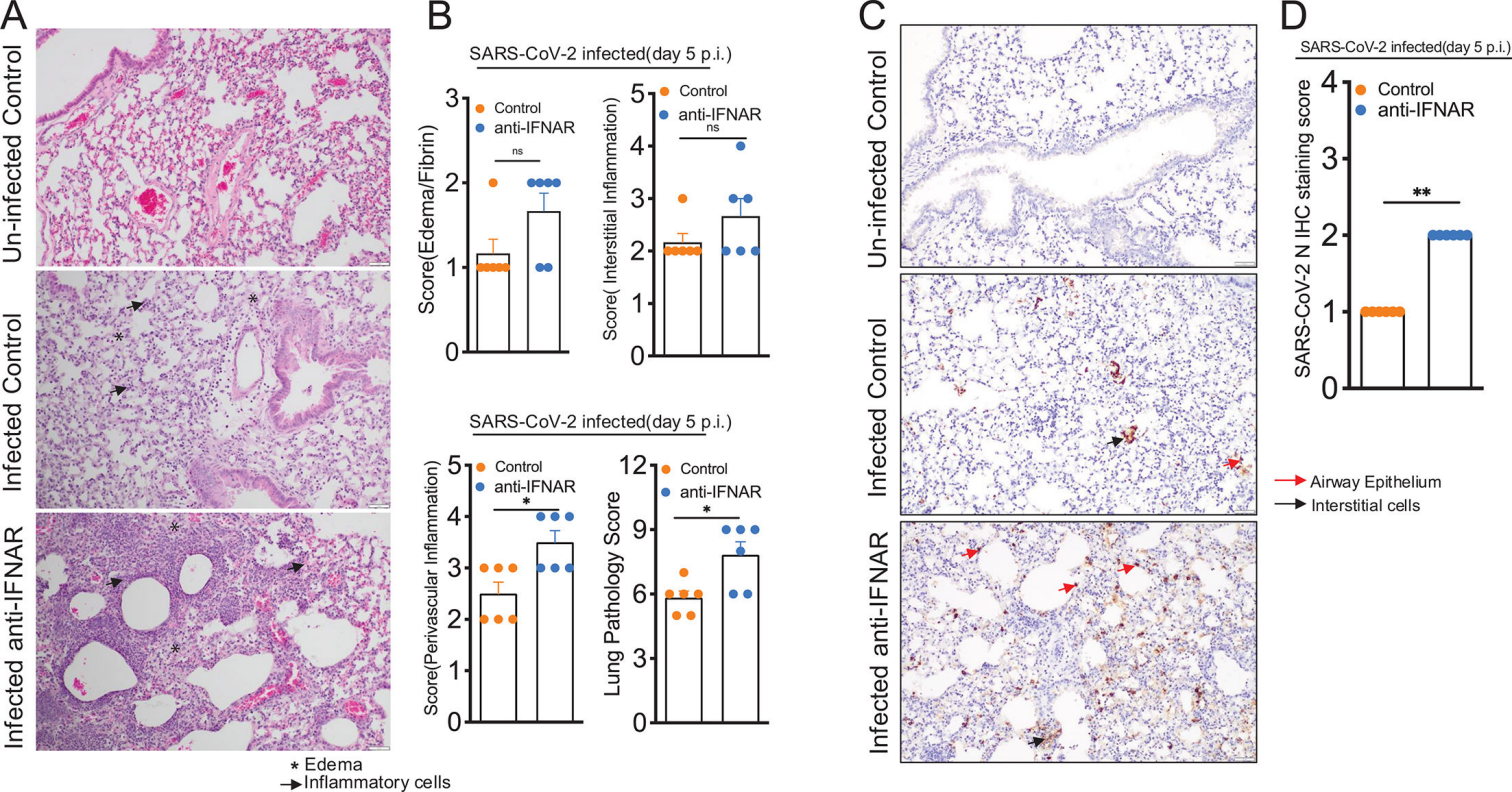
IFN-I receptor signaling protects mice from SARS-CoV-2-induced lung pathology. Twelve- to 14-week-old wild-type Balb/c mice were treated with control or anti-IFNAR antibody and infected intranasally with 250 PFU of MA-CoV-2. Mice were euthanized at 5 dpi. PBS-perfused lungs collected in Zn formalin were stained with hematoxylin and eosin to assess lung lesions. (**A**) Representative photomicrographs showing edema and inflammatory cell infiltration in the lungs. (**B**) Summary histology scores for edema/fibrin, interstitial inflammation, perivascular inflammation, and cumulative lung pathology score. (**C**) Immunohistochemical analysis for SARS-CoV-2 N antigen in lungs at 5 dpi. (**D**) Summary immunohistochemical scores for SARS-CoV-2 N antigen in lungs. Each point in the scatter plot represents individual mice (*n* = 6 for each group). Statistical significance was determined using Student’s *t*-test (**B**) and Mann–Whitney test (**D**) with *P < 0.05, **P < 0.01, and ***P < 0.001.

Mutations in genes related to IFN signaling, loss of the IFN response, and IFN-I autoantibodies are associated with severe COVID-19 in humans (71, 92, 93). However, because of the lack of IFN-deficient COVID-19 lung samples, the effect of IFN-I deficiency on SARS-CoV-2-induced lung inflammation and pathology is not well understood. To address these questions, we examined microscopic changes in the lungs of control and anti-IFNAR mAb-treated mice infected with MA-CoV-2. We observed evidence of organizing pneumonia with pockets of alveolar and perivascular inflammation characterized by polymorphonuclear neutrophil infiltration (Fig. 6A and B). We also observed increased edema and fibrin deposition in four out of the six IFNAR mAb-treated lungs compared with one out of the six control lungs (Fig. 6A and B). The collective lung pathology score (cumulative edema/fibrin, perivascular inflammation, and interstitial inflammation scores) was significantly greater for IFNAR-deficient lungs than for infected control lungs (Fig. 6B). We also immunostained lung tissues for SARS-CoV-2 N antigen and observed few viral antigen-positive cells in infected control lungs, whereas the viral antigen was abundant in IFNAR lungs, with antigen primarily detected in interstitial macrophages, pneumocytes, and isolated airway epithelial cells (Fig. 6C and D). Overall, these results showed that IFNAR signaling is essential for the suppression of viral replication and host protection during SARS-CoV-2 infection.

## DISCUSSION

CoVs possess a single-stranded RNA genome that activates TLR7/8 to induce antiviral IFN/ISG and robust inflammatory cytokine/chemokine responses (44, 46, 49, 50, 53, 54, 79–81, 84, 94). However, whether the TLR7/8-induced IFN/ISG response is sufficient to protect the host or if TLR7/8-induced robust inflammation causes lung pathology and severe disease is not clearly defined. In this study, we provided conclusive evidence that TLR7 signaling and TLR7-induced IFN/ISG responses are protective during SARS-CoV-2 infection in mice. These results corroborate SARS-CoV-2 studies in humans that revealed severe COVID-19 in people with TLR7 mutations/variants (44, 46, 53, 54, 79–81, 94). Moreover, the use of anti-IFNAR mAb treatment not only mimics COVID-19 patients with anti-IFN autoantibodies (60, 62, 95) but also provides conclusive evidence that anti-IFN/IFNAR antibodies promote severe disease upon SARS-CoV-2 infection. Our results also demonstrate the effects of TLR7 deficiency and impaired IFN/ISG responses on lung virus titers, antiviral/inflammatory responses, and pulmonary damage, all of which could not be evaluated in COVID-19 patients because of the lack of human autopsy or lung biopsy samples. Additionally, given the similar disease outcomes in SARS-CoV-2-infected controls and TLR7-deficient/mutant individuals and responses to anti-IFN/IFNAR antibodies in mice and humans, our results further support the use of a mouse model and mouse-adapted SARS-CoV-2 to study COVID-19 pathogenesis.

We show that TLR7 signaling is essential for a strong IFN/ISG response and for controlling SARS-CoV-2 replication in the lungs. However, these results do not identify a key cellular source(s) of IFNs in MA-CoV-2 lungs. Animal model studies and *in vitro* data from murine and human PBMCs challenged or treated with SARS-CoV-2, CoV RNA, or viral RNA mimics revealed that pDCs, alveolar macrophages (AMs), DCs, and mDCs are the primary sources of IFN-Is during CoV infection (44, 46, 53, 54, 79–81, 94). Notably, hematopoietic cells are not productively infected by hCoVs; therefore, it is likely that these cells produce IFNα/β via abortive infection or upon sensing CoV ssRNA released from infected epithelial cells (35–39, 96). In contrast to the cellular source of IFNα/β, monocyte–macrophages and neutrophils are the primary producers of inflammatory cytokines and chemokines (26, 85–90). In addition to hematopoietic cells, lung epithelial cells also express TLR7. However, the relative contribution of lung epithelial cells to SARS-CoV-2-induced antiviral and inflammatory responses is not known, as blocking epithelial cell TLR7 signaling with a specific inhibitor did not affect SARS-CoV-2-induced IFN or inflammatory responses (97). Conversely, we showed a reduced lung IFN response in MERS-CoV-infected TLR7-deficient mice transduced with adenovirus-5 encoding human dipeptidyl peptidase 4 (Ad5-hDPP4) (44). In these studies, although hDPP4 is expressed on airway and alveolar epithelial cells, transduction of hDPP4 on AMs and subsequent infection or stimulation of AMs with MERS-CoV to elicit an IFN/ISG response cannot be ruled out. Nonetheless, our results in the current manuscript show that pDC, but not AMs, are the primary sources of IFNs during SARS-CoV-2 infection, which is in agreement with SARS-CoV and SARS-CoV-2 studies (98–101). Therefore, both hematopoietic cell (pDC)- and lung epithelial cell-derived IFN/ISG responses likely control lung SARS-CoV-2 replication and inflammation. Our results demonstrate that the cellular source of IFN varies with type of human CoV, likely reflecting the ability of hCoVs to infect (productively or abortively) a cell type. We also show that compared to blocking IFNAR signaling, which shows 100% mortality, TLR7^−/−^ mice are less susceptible (~70% mortality). These results imply that the other innate immune sensors, such as RIG-I-like receptors (RLRs), primarily MDA5, might play an important role in IFN/ISG induction and host protection (96, 102).

Type I and III IFNs are protective during a variety of virus infections, and their protective efficacy is dictated by the timing and magnitude of the IFN response during hCoV infection. These conclusions are supported by recent studies, including ours, which showed that an early IFN response relative to peak virus replication is protective during CoV and influenza virus infection, whereas a delayed endogenous or exogenous IFN response causes severe disease (28, 44, 103, 104). Our studies also revealed CoV-specific effects of IFN response on disease outcomes, as demonstrated by the detrimental role of IFNs during SARS-CoV infection and, in contrast, a protective effect during mouse-CoV, MERS-CoV, and SARS-CoV-2 infection (28, 44, 46). While recent studies have shown enhanced susceptibility of IFNAR^−/−^ K18-hACE2 mice upon SARS-CoV-2 infection (105, 106), the results of our anti-IFNAR mAb treatment studies closely mimic the anti-IFN autoantibody response in patients with severe COVID-19 (59, 60, 62, 64, 65, 67, 69–71, 73). In addition to impaired IFN/ISG responses, excessive myeloid cell-mediated inflammatory cytokine responses cause severe disease in CoV-infected hosts. We and others have also shown that type I IFNs play differential roles in the recruitment of IMMs and neutrophils during RSV, SARS-CoV, MERS-CoV, and SARS-CoV-2 infections (28, 85, 107). In agreement with these results, low *Ifna*/*b* and *Ccl2* transcript levels correlated with a reduced frequency of IMM at 2 dpi in TLR7^−/−^ mice, although the total number of IMMs was unchanged. Conversely, type I IFNs suppress neutrophil recruitment (44), and as a result, reduced type I IFNs in TLR7^−/−^ mice likely facilitate neutrophil accumulation (Fig. 3) and neutrophil-mediated lung pathology. In this study, we have examined differences in key cell types, such as inflammatory monocytes, neutrophils, pDCs, and AMs, that play an important role in SARS-CoV-2-infected lungs. However, we cannot exclude the possible differences in innate lymphoid cells, although it is unlikely that a compensatory increase in innate lymphoid cells (which are usually protective) would cause severe disease. Robust myeloid cell accumulation and TLR7-mediated inflammation via MyD88-NF-κB/MAPK pathway-induced inflammatory cytokine and chemokine activities may promote severe disease in WT mice. However, given the reduced inflammatory cytokine levels in TLR7^−/−^ (Fig. 2), it is unlikely that myeloid cell-mediated NF-κB/MAPK-induced inflammatory mediators caused severe pneumonia in TLR7^−/−^ mice. In contrast, robust virus replication and virus- and myeloid cell-induced direct epithelial cell injury in these immunodeficient hosts may drive severe pneumonia.

TLR7-mediated IFN response correlates with virus clearance and host protection. Although we did not examine whether TLR7 deficiency leads to suboptimal adaptive immunity and thus impaired virus clearance, we believe that the adaptive immunity likely plays a minor role, if any, in virus clearance and disease outcome in TLR7^−/−^ mice. This statement stems from the fact that SARS-CoV-2 titer is significantly higher in TLR7^−/−^ lungs at both days 2 and 5 post-infection, at which time adaptive immunity plays a minor role. Furthermore, our results in Fig. 1B show that mortality in TLR7^−/−^ mice begins at day 5 post-infection, with the majority of TLR7^−/−^ mice succumbing to infection by days 8/9 post-infection. Published studies, including ours, show that T cell response peaks between days 8 and 10 post-infection, and antibody response during primary infection peaks between days 10 and 15 post-infection. Therefore, we believe that adaptive immunity plays a minor role in virus clearance and disease outcome in TLR7^−/−^ mice compared to controls.

Severe COVID-19 lungs show diffuse alveolar damage, lung edema, fibrin deposition, and the accumulation of inflammatory cells (108, 109). In agreement with these results and several other published works, our data from control infected lungs revealed increased alveolar edema/fibrin deposition and excessive myeloid cell accumulation (Fig. 4 and 6). Despite extensive lung pathology data from deceased COVID-19 patients (109–111), due to the lack of autopsy and lung biopsy samples from TLR7- and IFN-deficient patients, the distribution of pulmonary lesions and SARS-CoV-2 antigens in the lungs of TLR7- and IFN-mutant/deficient COVID-19 patients is not known. Here, we demonstrated greater alveolar edema/fibrin deposition, interstitial inflammation and thickening, and perivascular inflammation in TLR7- and IFNAR-deficient lungs than in WT control lungs (Fig. 4 and 6). Additionally, blocking IFNAR activity caused organizing pneumonia (Fig. 6). These results provide insight into potential changes in the lungs of COVID-19 patients with TLR7 and IFN deficiency/mutation. We show increased viral antigen levels in lung epithelial cells and macrophages of TLR7-deficient mice compared to naïve and control-infected lungs. While increased SARS-CoV-2 N antigen levels in lung epithelial cells are likely due to reduced IFN/ISG response, the same might not be true for increased N antigen in lung macrophages, as these cells are not permissive to SARS-CoV-2 infection (38, 112), and if infected, it is a non-productive infection. Therefore, phagocytosis of viral particles or viral antigen released from infected dead epithelial cells likely contributes to the high abundance of N protein in the macrophages. This does not necessarily mean that macrophages in TLR7^−/−^ mice have an increased phagocytic ability on a per-cell basis. Instead, it is likely that in TLR7^−/−^ mice, increased amounts of viral antigens or particles are available for each macrophage to phagocytose. Collectively, our results further revealed that severe lung pathology and fatal pneumonia in TLR7- and IFN-deficient mice correlate with increased lung virus titers, viral antigen abundance, and inflammatory cell accumulation (Fig. 4 and 6), all of which collectively may contribute to severe pneumonia and fatal disease in these hosts.

In summary, our results showed that TLR7 signaling is protective during SARS-CoV-2 infection in mice, similar to that observed in humans with COVID-19. Our results further revealed that enhanced virus replication, virus-induced direct cytopathic effects, and neutrophil-mediated inflammation likely cause lung pathology and severe pneumonia in TLR7^−/−^ mice compared to that observed in WT control mice. Moreover, given the similar disease outcomes in TLR7- and IFNAR-deficient humans and mice, we showed that MA-CoV-2-infected mice serve as a reliable model for studying COVID-19 pathogenesis and the antiviral response.

## Data Availability

All relevant data supporting the findings of this study are available within the article and its supplemental material. Detailed replicate information for representative data, along with the raw data files, will be provided upon request from the corresponding author.

## SUPPLEMENTAL MATERIAL

Figure S1 jvi.01668-24-s0001.eps pDCs are a major contributor of type I interferon in SARS-CoV-2-infected lungs.

Figure S2 jvi.01668-24-s0002.eps TLR7 deficiency causes differential myeloid cell accumulation in SARS-CoV-2-infected lungs.

ASM does not own the copyrights to Supplemental Material that may be linked to, or accessed through, an article. The authors have granted ASM a non-exclusive, world-wide license to publish the Supplemental Material files. Please contact the corresponding author directly for reuse.
